# Lipocalin-2 negatively regulates epithelial–mesenchymal transition through matrix metalloprotease-2 downregulation in gastric cancer

**DOI:** 10.1007/s10120-022-01305-w

**Published:** 2022-06-15

**Authors:** Sadaaki Nishimura, Yurie Yamamoto, Atsushi Sugimoto, Shuhei Kushiyama, Shingo Togano, Kenji Kuroda, Tomohisa Okuno, Hiroaki Kasashima, Masaichi Ohira, Kiyoshi Maeda, Masakazu Yashiro

**Affiliations:** 1 Molecular Oncology and Therapeutics, Osaka Metropolitan University Graduate School of Medicine, 1-4-3, Asahi-machi, Abeno-ku, Osaka City, Osaka 545-8585 Japan; 2 Department of Gastroenterological Surgery, Osaka Metropolitan University Graduate School of Medicine, Osaka, Japan; 3 Cancer Center for Translational Research, Osaka Metropolitan University Graduate School of Medicine, Osaka, Japan

**Keywords:** Lipocalin-2, Epithelial–mesenchymal transition, Gastric cancer

## Abstract

**Background:**

Although the role of Lipocalin-2 (LCN2) in cancer development has been focused on recent studies, the molecular mechanisms and clinical relevance of LCN2 in gastric cancer (GC) still remain unclear.

**Methods:**

Transcriptome analysis of GC samples from public human data was performed according to Lauren’s classification and molecular classification. In vitro, Western blotting, RT-PCR, wound healing assay and invasion assay were performed to reveal the function and mechanisms of LCN2 in cell proliferation, migration and invasion using LCN2 knockdown cells. Gene set enrichment analysis (GSEA) of GC samples from public human data was analyzed according to LCN2 expression. The clinical significance of LCN2 expression was investigated in GC patients from public data and our hospital.

**Results:**

LCN2 was downregulated in diffuse-type GC, as well as in Epithelial–Mesenchymal Transition (EMT) type GC. LCN2 downregulation significantly promoted proliferation, invasion and migration of GC cells. The molecular mechanisms of LCN2 downregulation contribute to Matrix Metalloproteinases-2 (MMP2) stimulation which enhances EMT signaling in GC cells. GSEA revealed that LCN2 downregulation in human samples was involved in EMT signaling. Low LCN2 protein and mRNA levels were significantly associated with poor prognosis in patients with GC. LCN2 mRNA level was an independent prognostic factor for overall survival in GC patients.

**Conclusions:**

LCN2 has a critical role in EMT signaling via MMP2 activity during GC progression. Thus, LCN2 might be a promising therapeutic target to revert EMT signaling in GC patients with poor outcomes.

**Supplementary Information:**

The online version contains supplementary material available at 10.1007/s10120-022-01305-w.

## Introduction

Gastric cancer (GC) is the fourth most common cause of death due to malignant diseases worldwide [1]. The prognoses of patients with unresectable advanced or recurrent gastric cancer remain particularly poor [2, 3]. Notably, diffuse-type gastric cancer (DGC), according to Lauren classification, shows poor clinical outcome with few targeted treatment options, rapid tumor progression and high metastatic ability compared to intestinal-type gastric cancer (IGC) [3, 4]. DGC is molecularly associated with epithelial–mesenchymal transition (EMT) signaling, which is one of the pathways to develop tumor progression and spread, resulting in poor prognosis in patients with GC [5, 6]. Although EMT-related upregulated proteins in GC have been studied extensively, their negative regulation has been limited to be investigated so far. Thus, understanding the negative regulatory mechanisms in this signaling pathway is crucial for improving the poor outcome of patients with GC.

Lipocalin-2 (LCN2), also known as neutrophil gelatinase-associated lipocalin, has emerged as a critical iron protein that blocks bacterial growth under physiological and inflammatory conditions [7, 8]. Recently, the oncological role of LCN2 has been investigated in several types of cancer and has been identified as being negatively associated with EMT signaling in cancer development [9–12]. However, the role of LCN2 in invasion and metastasis during GC progression has not been clarified.

Here, we investigated the potential role of LCN2 in GC cell proliferation and invasion in vitro and subsequently validated these observations using human datasets. We found that EMT signaling was downregulated by LCN2 in GC cells. There is a correlation between LCN2 expression and the loss of EMT characteristics in GC patients. At the mechanistic level, LCN2 negatively regulate Matrix Metalloproteinases-2 (MMP2), leading to EMT inactivation.

## Materials and methods

### Clinical data from public database

To clarify the molecular characteristics of diffuse-type gastric cancer (DGC) and intestinal-type gastric cancer (IGC) based on the Lauren classification, human gene expression profiling for gastric cancer was obtained from the Gene Expression Omnibus database (GSE113255)[13], which was generated by RNA-seq based transcriptome analysis of Korean patients with DGC (*n* = 107) and IGC (*n *= 23). The Asian Cancer Research Group (ACRG) cohort study from GEO database (GSE62254) [5] and The Cancer Genome Atlas (TCGA) cohort study from cBioportal for Cancer Genomics (http://www.cbioportal.org/) were also evaluated for genomic profiles and clinical data of patients with gastric cancer.

### Bioinformatics

All raw FASTQ files obtained from GSE113255 were subjected to pseudo-alignment using Salmon v0.12.0 [14] to quantify the transcript expression based on GRCh38. Sequence reads were also aligned to GRCh38 using HISAT2 version 2.1.0 [15] and visualized using IGV version2.6.3 [16] to explore genomic alterations. Transcripts-per-million (TPM) counts were derived using the tximport R package [17]. Gene expression data were analyzed to evaluate significantly differentially expressed gene (DEG) using DESeq2 R package [18], as follows: false discovery rate (FDR) < 0.1 and log2 fold change > 2 was designed as the threshold. DEG was uploaded to STRING database (https://string-db.org/) to detect the interaction of proteins encoded by DEG. Gene matrix files obtained from GSE62254 as well as TCGA cohort study was used as input file for Gene Set Enrichment Analysis (GSEA). GSEA was performed using GSEA 4.2.3 software with 1000 gene-set permutations using the gene-ranking metric T-test with the collections h.all.v7.5.1.symbols (Hallmarks). The “*LCN2* high” and “*LCN2* low” in GSEA are based on its Z-score.

### Cell lines

OCUM-12 [19] cells were established in our laboratory, and NUGC-3, MKN-45 and MKN-74 cells were obtained from the JCRB Cell Bank (Osaka, Japan). A total of four gastric cell lines were incubated in a culture medium consisting of Dulbecco’s modified Eagle’s medium (DMEM; Nikken, Kyoto, Japan) with the addition of 10% fetal bovine serum (Nichirei, Tokyo, Japan), 100 IU/mL penicillin (Wako, Osaka, Japan), 100 mg/mL streptomycin (Wako), and 0.5 mmol/L sodium pyruvate (Wako). All the cell lines used in this study were authenticated by STR profiling before distribution. We tested for mycoplasma contamination in the cell lines and proved that there was no mycoplasma contamination in the cell lines. Cells were cultured at 37 °C in 21% O_2_, whereas OCUM12 hypoxic cells were cultured at 37 °C in 2% O_2_.

### siRNA for knockdown of LCN2 expression

LCN2 siRNA#1 (siLCN2#1), LCN2 siRNA#2 (siLCN2#2) and control siRNA (siControl) were transfected into OCUM-12 and NUGC-3 cells using Lipofectamine RNAiMAX (Invitrogen) according to the manufacturer’s protocol. The sequences of siLCN2#1 were as follows: 5’-GCAUGCUAUGGUGUUCUUCTT-3’ (forward) and 5’-GAAGAACACCAUAGCAUGCTG-3’, as previously reported [11]. siLCN2#2 (ID: s8112) and siControl were purchased from Ambion (Life Technologies, Carlsbad, CA, USA). The knockdown efficacy was evaluated forty-eight hours after transfection.

siLCN2#2 was mainly used in subsequent experiments using OCUM-12 cells because of knockdown efficacy at LCN2 protein level, whereas siLCN2#1 was mainly used in those of NUGC-3 cells to avoid siRNA off-target effect.

### RT-PCR and quantitative real-time PCR

Total RNA from GC cells was isolated using TRIzol™ procedure. Total RNA was purified and quantified using the NanoDrop ND-1000 spectrophotometer (NanoDrop Technologies, Wilmington, DE). RT-PCR was performed using the OneStep RT-PCR kit (QIAGEN, Hidden, Germany), according to the manufacturer’s protocol. Quantitative real-time PCR (qPCR) was performed using ABI Prism 7000 (Applied Biosystems, Foster City, CA, USA) with THUNDERBIRD® SYBR® qPCR Mix (TOYOBO, Osaka, Japan). The amplification parameters were set at 95 °C for 15 s and 60 °C for 60 s (40 cycles total). The mRNA level of each gene was normalized by the internal control GAPDH. The primers are described in Table S1.

### Capillary sequencing analysis

DNA templates derived from RT-PCR were sequenced using BIG Dye terminators (version 3.1). PCR products were analyzed using a genetic analyzer (ABI PRISM 3130xl; Applied Biosystems, Foster City, CA) according to the manufacturer^’^s instructions.

### Proliferation assay

The effect of LCN2 knockdown on the proliferation of GC cells was determined by the MTT assay (Sigma-Aldrich, St Louis, MO, USA) assay. A total of 1 × 10^4^ GC cells were seeded into 96-well plates with a culture medium exposed to siLCN2 for 48 h.

### Western blot analysis

OCUM-12, NUGC-3, MKN-45, and OCUM-74 cells were rinsed with PBS and incubated in DMEM. After incubation, the cell extracts (20 mg protein) were used for western blot analysis. The following primary antibodies were used: β-actin (1:3000; Sigma-Aldrich, St Louis, MO, USA), LCN2 (1:2000; Proteintech, Rosemont, IL, USA), MMP2 (1:1000; Abcam, Cambridge, MA, USA) and MMP9 (1:500; Santa Cruz, Dallas, TX, USA).

### Morphological changes

Cancer cells were cultured under normal conditions 24hs after transfection with siControl or siLCN2 and cell morphology was observed microscopically. EMT was determined when polygonal or spindle-shaped cancer cells were found by phase-contrast microscopy.

### Wound healing assay

Twenty-four hours after transfection with siControl or siLCN2, GC cells were cultured in 96-well plates (Essen ImageLock; Essen Instruments, Birmingham, UK) and allowed to generate a wound in the cell monolayer using a 96-well WoundMaker (Essen Bioscience, Ann Arbor, MI, USA). Cancer cells were cultured in DMEM with 5% FBS. Images of the scratched fields were captured every 2 h by IncuCyte live-cell imaging system and software (Essen Instruments). The degree of cell migration was analyzed at 24 h, 48 h after wound treatment as a percentage of wound confluence.

### Invasion assay

The in vitro invasiveness was measured by a two-chamber Matrigel invasion assay involving chemotaxis cell chambers (Millipore, Billerica, MA, USA) with a 12-μm pore membrane filter coated with 50 µg of Matrigel (upper chamber) in a 24-well culture plate (lower chamber), as previously reported [20]. GC cells (1 × 10^4^ cells per 200 μL per chamber) were seeded in the upper chamber, and 500 μL of DMEM with 2% FBS was added to the lower chamber. Seventy-two hours after incubation, cancer cells that invaded the lower surface of the membrane through a filter were stained with Diff-Quik (Sysmex, Kobe, Japan) and were manually counted under a microscope at × 200 magnification. Six randomly chosen fields were counted in each group.

### Immunohistochemical analysis

Immunohistochemical staining was performed using LCN2 antibody (Proteintech, Rosemont, IL, USA). Immunohistochemical analysis of LCN2 was performed as described below. In brief, we performed deparaffinization and slides were heated for 10 min at 105 °C in an autoclave in Target Retrieval Solution (Dako). After blocking the endogenous peroxidase activity, the specimens were incubated with LCN2 antibody (1:150) for 1 h at room temperature. The slides were incubated with biotinylated anti-mouse IgG for 10 min. The slides were treated with streptavidin-peroxidase reagent and counterstained with Mayer’s hematoxylin. LCN2 expression was analyzed by staining intensity and the percentage of stained cancer cells as follows. 0 = no staining or weak staining in less than 40% of cancer cells; 1 +  = weak staining in more than 40% of cancer cells or strong staining in less than 40% of cancer cells; 2 +  = strong immunostaining in more than 40% of cancer cells. Scores of 0 were assigned to the LCN2-low expression group, whereas scores of 1 + or 2 + were assigned to the LCN2-high expression group. The evaluation was performed by two double-blinded independent observers who were unaware of the clinical data and outcome.

### Statistical analysis

All analyses were performed using GraphPad Prism 9.2.0 (GraphPad Software, La Jolla, CA, USA). In vitro data are expressed as the mean ± standard deviation and significant differences were analyzed by the unpaired Student’s t test. Correlations between the high and low LCN2 expression groups were determined using the chi-square test. Differences between more than three groups were determined using one-way ANOVA test (parametric). Survival rates were estimated using the Kaplan–Meier method, and differences in survival according to the group classification of patients were analyzed by log-rank test. Multivariate analyses were performed to determine the significant prognostic factors using Cox regression models. Differences were considered statistically significant at *p* < 0.05.

## Results

### LCN2 is downregulated in diffuse-type gastric cancer associated with EMT activation

**Fig. 1:**
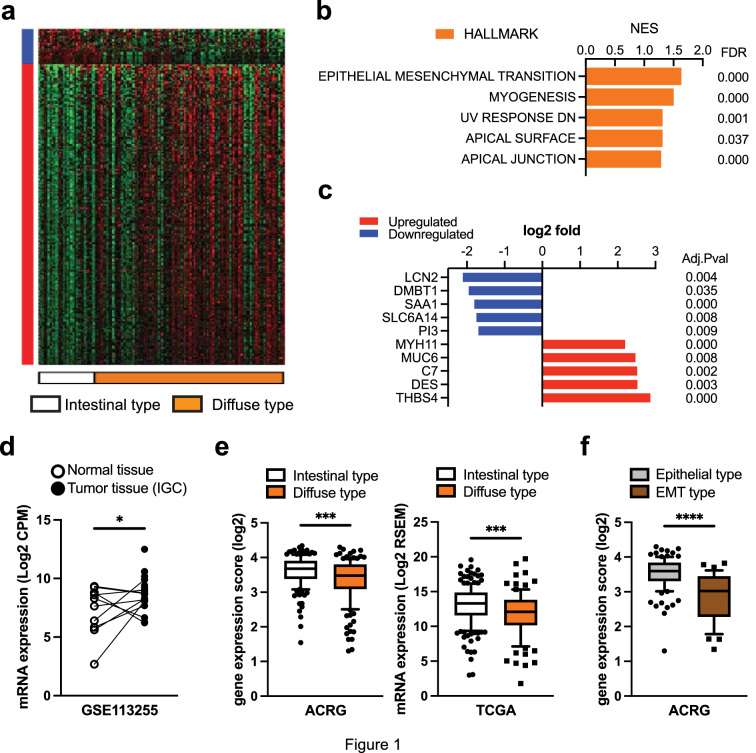
Transcriptome analysis of gastric cancer patients from public data. **a** Heatmap of RNA-seq data from 130 patients with gastric cancer according to Lauren classification representing significant differential expressed gene. Blue bar indicates upregulated genes. Red bar indicates downregulated genes. **b** GSEA of transcriptome data from RNA-seq of DGC patients as compared to IGC patients using HALLMARK gene sets. FDR, false discovery rate. NES, normalized enrichment score. **c** The top five upregulated and downregulated genes in DGC samples as compared to IGC samples from GSE 113,255. Adj Pval, adjusted p-value. **d** LCN2 mRNA expression levels in IGC tissues (n = 23) and paired corresponding normal tissues (n = 10). **e** LCN2 mRNA expression levels in DGC patients compared with IGC patients in ACRG (n = 292) and TCGA (n = 294) cohort studies. **f** LCN2 mRNA expression level in DGC patients from ACRG cohort study (n = 146) according to molecular classification. EMT, Epithelial–Mesenchymal Transition. The results are presented as a 10–90 percentile plot. *p < 0.05, **p < 0.01, ***p < 0.001, ****p < 0.0001

Based on bioinformatics analysis from RNA-seq data of GSE113255, a total of 320 genes were identified as upregulated DEGs in patients with DGC, whereas a total of 42 genes were identified as downregulated DEGs in those with DGC (Table S2). Figure 1a illustrates the heat map of DEGs from GSE113255. Gene set enrichment analysis (GSEA) of DEGs demonstrated that gene sets related to epithelial–mesenchymal transition (EMT) and myogenesis were significantly upregulated in DGC, in keeping up with a common characteristic feature of DGC in previous reports [5, 21] (Fig. 1b). Subsequently, we focused on the top five upregulated and downregulated genes in patients with DGC according to log2 fold. In top 5 upregulated genes, it is well known that *MYH11* and *DES* gene-encoded myosin-11 and desmin as an EMT marker. Likewise, *Thrombospondin-4* (*THBS4*) was one of the most upregulated genes in DGC (Fig. 1c), which was correlated with cancer-associated fibroblasts in GC to stimulate EMT, as we previously reported [22]. In contrast, patients with DGC in GSE113255 dataset showed downregulation of *Lipocalin-2* (*LCN2*) expression compared with patients with IGC. Further assessment revealed that LCN2 mRNA levels in IGC were significantly higher than those in matched normal tissues (Fig. 1d). To determine whether this Korean cohort study is consistent with previous gene expression data, bioinformatics analyses of human gastric cancer datasets (ACRG, TCGA) were performed and revealed that LCN2 mRNA expression was significantly reduced in DGC as compared to IGC (Fig. 1e). Furthermore, the expression level of LCN2 was significantly higher in epithelial type DGC than in EMT type GC, which was recognized as a distinct subtype of GC and showed impaired survival in the ACRG cohort study (Fig. 1f). Therefore, we hypothesized that LCN2 encoded by *LCN2* play a critical role in GC progression and might be negatively associated with the mechanisms of EMT in GC.

### LCN2 downregulation in GC cells promotes the proliferation and migration ability

**Fig. 2:**
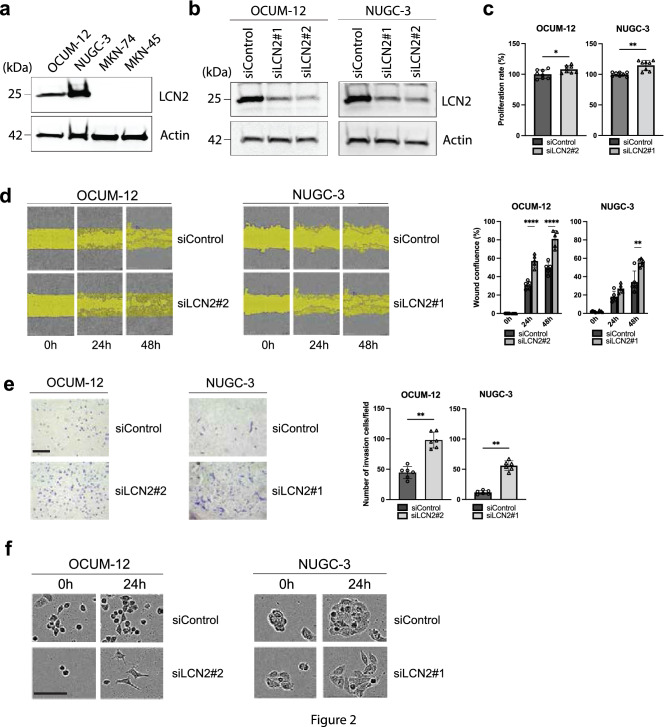
Downregulation of LCN2 induces cell proliferation, migration, invasion and EMT phenotype. **a** Endogenous LCN2 were expressed by western blot analysis in the gastric cancer cell line. **b** The efficiency of LCN2 knockdown using two different siRNAs was analyzed in OCUM-12 and NUGC-3 cells by western blot analysis. **c** The viability of OCUM-12 and NUGC-3 cells was measured by MTT assay 48 h after siRNA transfection (n = 8). **d** Representative Images of cell migration in OCUM-12 and NUGC3 cells at different time points after scratching. LCN2 knockdown and control cells were analyzed. Wound confluency was quantified (n = 6). **e** Representative image of invading OCUM-12 and NUGC-3 cells using a two-chamber Matrigel invasion assay. LCN2 knockdown and control cells were analyzed. The number of cells were counted (n = 6). **f** The number of polygonal or spindle-shaped cells, indicating epithelial–mesenchymal transition (EMT), increased in both OCUM-12 and NUGC-3 cells after siLCN2 treatment. Scale bar, 100 μΜ. Results are presented as mean ± SD. *p < 0.05, **p < 0.01, ***p < 0.001, ****p < 0.0001 compared with siControl

To identify a biological role of LCN2 in GC cells, we used three cell lines (OCUM-12, NUGC-3 and MKN-45) derived from DGC and one cell line (MKN-74) derived from IGC to examine the expression level of LCN2 by western blot analysis and RT-PCR. As shown in Fig. 2a and Figure S1a, LCN2 was remarkably expressed in OCUM-12 and NUGC-3 cells as confirmed by western blot analysis and RT-PCR. In contrast, the expression of LCN2 was loss in MKN-74 and MKN-45 cells. In this context, OCUM-12 and NUGC-3 cells were transfected with siLCN1#1, siLCN2#2 and siControl as negative controls. It is clear that the expression level of LCN2 was reduced by siLCN2 in these cells, as determined by Western blotting, RT-PCR and qPCR (Fig. 2b, S1b and S1c). The effect of LCN2 downregulation on the proliferation of OCUM-12 and NUGC3 cells is shown in Fig. 2c. LCN2 knockdown significantly stimulated the growth of OCUM-12 and NUGC-3 cells in vitro. Figure 2d shows a representative phase-contrast image of the wound-healing assay. The number of migrating OCUM-12 and NUGC-3 cells was significantly increased by *LCN2* knockdown (*P* < 0.0001 and *P* < 0.01, respectively). Figure 2e provides a representative phase-contrast image of OCUM-12 and NUGC-3 that invaded a 12 µm pore membrane filter. The number of invading cancer cells was significantly increased after transfection with siLCN2 in OCUM-12 and NUGC-3 cells in comparison with siControl. Moreover, *LCN2* knockdown cells showed a more polygonal or spindle-shaped morphology than control cells, resulting from EMT in GC cells (Fig. 2f). Altogether, these findings suggest that LCN2 in GC cells negatively induces the EMT phenotype which enhances cancer cell proliferation and invasion.

### LCN2 negatively regulates EMT pathway through MMP2 inactivation

**Fig. 3:**
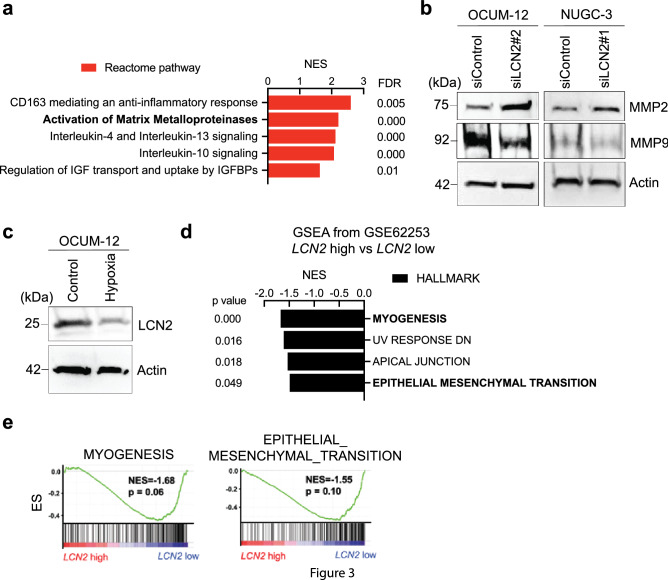
LCN2 inactivation is associated with EMT signaling via MMP2 activation. **a** LCN2 related pathway was analyzed by Reactome analysis. FDR, false discovery rate. NES, normalized enrichment score. IGF, Insulin-like Growth Factor; IGFBPs, Insulin-like Growth Factor Binding Proteins. **b** Western blot analysis revealed several types of Matrix Metalloproteases in OCUM-12 and NUGC-3 cells transfected with siLCN2 and siControl, respectively. **c** The expression level of LCN2 was significantly decreased under hypoxic condition in OCUM-12 cells. **d** GSEA of transcriptomic data of GC patient sample from GSE 62,254 using HALLMARK gene sets and stratified on the basis of *LCN2* expression. **e** GSEA plot of “MYOGENESIS”, “EPITHELIAL_MESENCHYMAL_TRANSITION” in the transcriptomic data of GC patient samples from TCGA using HALLMARK gene sets and stratified on the basis of *LCN2* expression

To determine how the EMT pathway is negatively regulated by LCN2 in GC cells, we performed a Reactome analysis using STRING. Reactome analysis showed that a crucial pathway in LCN2 is involved in “Activation of Matrix Metalloproteinases” signaling (Fig. 3a). Based on this result, the expression of Matrix Metalloproteinases in *LCN2* knockdown GC cells was analyzed to elucidate the mechanism of EMT stimulation. Importantly, MMP2 expression was significantly increased by LCN2 downregulation, whereas MMP9 levels were reduced by LCN2 knockdown in GC cells (Fig. 3b). We next sought to establish the association between LCN2 and EMT markers. The expression level of LCN2 was additionally examined in OCUM-12 cells under hypoxic conditions (OCUM-12/hypo), which was closely associated with EMT activation as we previously reported [19, 23]. Interestingly, OCUM-12/hypo cells showed a strong decrease in LCN2 expression compared with OCUM-12 cells under normoxic conditions (Fig. 3c). Moreover, MMP2 was activated to a greater extent in OCUM-12/hypo cells [19]. To validate these observations in human patients, we investigated whether low expression of LCN2 was inversely correlated with EMT pathway signature in human datasets. GSEA of ACRG dataset from 292 patients with GC [5] using the HALLMARK gene set compilation showed that *LCN2* expression negatively correlated with “Myogenesis”, “UV_Response_DN”, “Apical_Junction”, “Epithelial_Mesenchymal_Transition” signatures (Fig. 3d). Similarly, the in-silico analysis of RNA-seq data of GC patients in the TCGA dataset also revealed a negative correlation between *LCN2* expression and “Myogenesis”, “Epithelial_Mesenchymal_Transition” signatures (Fig. 3e). Taken together, these findings establish that *LCN2* downregulation is associated with an increment in the EMT pathway through MMP2 activation in GC cells and human patients.

### Interaction of LCN2 with SAA1 in GC cells and patients.

**Fig. 4:**
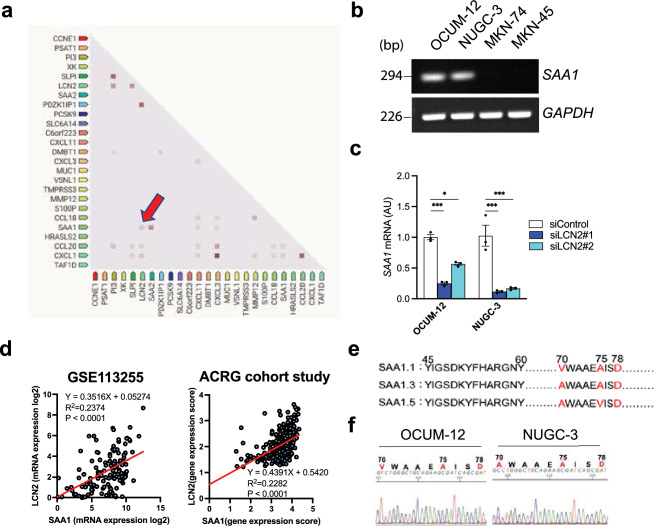
Association between LCN2 and SAA1 in GC cells and patients. **a** STRING predicted protein interactions derived from significantly downregulated DEG in GES113255. LCN2 interaction with SAA1 was targeted in this study (red arrow). **b** SAA1 mRNA level was detected by RT-PCR in gastric cancer cell lines. **c** qPCR analysis of mRNA of SAA1 in OCUM-12 and NUGC-3 cells transfected by siRNA targeted to *LCN2* (n = 3). **d** A positive association of mRNA levels between LCN2 and SAA1 was detected in GC patients from GSE113255 and ACRG cohort studies. **e** *SAA1* phenotypes are distinguished by different amino acids in each of the variants, which are highlighted in red. **f** Sanger sequencing of *SAA1* in OCUM-12 and NUGC-3 cells. Amino acid changes at positions 70, 75 and 90 are highlighted in red. Results are presented as mean ± SD. *p < 0.05, **p < 0.01, ***p < 0.001, ****p < 0.0001 compared with siControl

To further assess the mechanisms of LCN2 in GC cells and patients, downregulated proteins encoded by DEG in patients with DGC from GSE113255 were analyzed to detect protein interaction with LCN2 by STRING. Among the 42 DEGs downregulated in patients with DGC, 25 of them encode proteins. Importantly, LCN2 is associated with Serum Amyloid A1 (SAA1) encoded by *SAA1*, which was top third most downregulated gene following *LCN2* and *DMBT1* in GSE113255 (Fig. 4a). Based on this result, RT-PCR was carried out to show the expression level of *SAA1* in the four GC cell lines, confirming a similar expression to that of *LCN2* (Fig. 4b). Moreover, *SAA1* expression was reduced in GC cells with LCN2 knockdown, demonstrating that the *SAA1* reduction was accompanied by *LCN2* downregulation (Fig. 4c and S2a). Bioinformatics analyses of human GC datasets revealed that there was a positive correlation between *LCN2* and *SAA1* expression in the tumors of GC patients (Fig. 4d). Lung et al. [24] demonstrated that *SAA1* allelic variants, such as *SAA1.1* and *SAA1.3*, can inhibit tumor metastasis by inhibiting angiogenesis in vitro* and *in vivo (Fig. 4e). Interestingly, the DNA sequences of OCUM-12 and NUGC-3 identified *SAA1* as *SAA1.1/SAA1.1* and *SAA1.1/SAA1.3*, respectively (Fig. 4f). Moreover, these polymorphisms accounted for over 70% of GC patients in the cohort study (GSE113255) (Figure S3a). Collectively, these results suggest that LCN2 deletion induces SAA1 hypoactivation, which is assumed to lead to tumor angiogenesis in GC based on the role of SAA1 polymorphisms.

### LCN2 low expression is correlated with impaired survival in clinical patients with gastric cancer

**Fig. 5:**
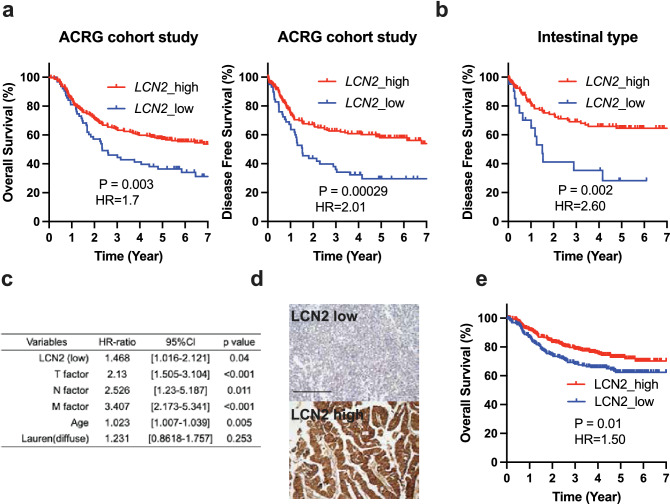
Clinical relevance of LCN2 in GC patients. **a** Kaplan–Meier curves for 7-year overall survival and disease-free survival of GC patients from ACRG cohort study according to *LCN2* expression (*n* = 292 and 275, respectively). *HR* hazard ratio. **b** Disease-free survival of IGC patients from the ACRG cohort study stratified on the basis with *LCN2* expression (n = 139). **c** Multivariate Cox multiple regression analysis for overall survival of GC patients in ACRG cohort study (n = 292). **d** LCN2 staining using the LCN2 antibody was mainly found in the cytoplasm of the cancer cells. All fields were analyzed at a magnification of × 200. Scale bar, 100 μΜ. **e** Overall survival of 590 patients with GC according to LCN2 expression by immunohistochemistry

We analyzed patients with GC in ACRG study according to the mRNA expression status of *LCN2* using Z-score. *LCN2* low expression was significantly correlated with T factor compared to high *LCN2* expression (Table S3). Figure 5a shows that the overall and disease-free survival of patients with LCN2 low expression was significantly poorer than that of patients with high *LCN2* expression (p = 0.003 and p < 0.0001, respectively). Interestingly, *LCN2* low expression was significantly associated with poor disease-free survival in patients with IGC from the ACRG cohort study (p = 0.002) (Fig. 5b). Furthermore, multivariate analysis revealed that *LCN2* low expression status was a significant adverse prognostic factor for overall survival of GC patients (p = 0.04), as well as tumor invasion and lymph node metastasis (Fig. 5C). TCGA cohort study also showed a tendency to be associated with *LCN2* expression in GC patients and overall survival (Figure S4a). To evaluate the significance of not only mRNA level but also protein level of LCN2 expression in GC, we next stained 590 GC samples taken by gastrectomy at our hospital, which showed that 37.9% of these cases are having the LCN2 low expression status. LCN2 low expression in GC was associated with T status and worse prognosis in terms of overall survival (Fig. 5d and 5e). Moreover, LCN2 protein levels in these samples were negatively correlated with T status, nodal involvement and M factor (Table S4). These results suggest that LCN2 expression is negatively associated with GC progression and metastasis.

## Discussion

Several gene expression datasets of GC cells and tissues have defined the genomic landscape of GC, and they have detected a new classification of GC that allows for the predicting of the developing novel diagnostic and prognostic markers [5, 13, 21]. LCN2 has emerged as an upregulated protein in various types of cancers and has been identified as a potential therapeutic target in previous studies [9, 11, 12, 25, 26]. However, the potential benefit of targeting LCN2 has not been applied to any cancer because of contradictory reports about the role of LCN2 [9–12, 25–28]. In this study, we showed that LCN2 mRNA levels in IGC tissues were higher than those in the corresponding normal tissues. In addition, LCN2 mRNA levels in DGC are lower than those in IGC. Notably, subgroup analysis demonstrated that *LCN2* expression was remarkably decreased in EMT type GC compared to that in epithelial type GC. These findings suggest that the role of LCN2 depends on the epithelial phenotype of GC. Interestingly, liver and ovarian cancer cells with EMT phenotype showed reduced LCN2 expression, while cells with epithelial phenotype had a strong expression of LCN2, which was concordant with our results [9, 10]. Collectively, LCN2 expression develops gastric tumors associated with the epithelial phenotype and is reduced as the tumor progresses, ending up being undifferentiated.

In this regard, we also demonstrated that *LCN2* expression was negatively correlated with DFS in patients with IGC, which is closely associated with epithelial type GC. Similarly, Lim et al. showed that LCN2 expression in ovarian cancer was positively correlated with early stages of ovarian cancer [10]. Although the mechanisms by which LCN2 regulates tumor progression in IGC are still unclear, LCN2 may be a potential molecular marker to monitor the transition from epithelial phenotype to EMT phenotype in IGC.

LCN2 expression is negatively associated with EMT signaling, which has been reported in several cancer type [9–12]. Our aim of this study was to determine the mechanisms by which LCN2 negatively regulates EMT signaling in GC. Previous studies reported that LCN2 downregulation led to upregulation of Twist1 and Snail, which are EMT-inducing transcription factors [9, 12]. We found a similar effect for OCUM-12/hypo cells [19, 23]. Moreover, in silico analysis of protein interactions predicted the interaction of LCN2 with MMP2, which was negatively controlled by LCN2 in this study. On the other hand, LCN2 regulates MMP9 in this study, which is concordant with previous report [29]. MMP2 and MMP9 activation has been implicated in tumor invasion and metastasis in GC [29, 30]. Therefore, the effect of LCN2 on cell invasion and migration was mediated by the suppression of MMP2 expression rather than MMP9 expression and subsequent inactivation of EMT signaling. GSEA analysis in human datasets also supports our hypothesis, suggesting that LCN2 is a strong negative marker for EMT signaling in GC, in spite of the difference between cancer cells within a single tumor.

The clinical significance of the expression level of LCN2 in GC has been uncertain so far. In this study, we clarified that LCN2 expression in GC was significantly associated with clinical outcome. However, it has been reported that tumor progression in other types of cancers is associated with LCN2 high expression rather than low expression, which is inconsistent with our results [25, 28]. These differences could be explained by differences in the SAA1 genotype or expression. The role of SAA1, accompanied by LCN2 expression, was predicted to lead to tumor suppression in this study. On the other hand, SAA1 phenotype, such as *SAA1.5,* has no tumor-suppressive effect [24]. Interestingly, there are significant differences in SAA1 genotype ratios between nasopharyngeal carcinoma [24] and GC (Figure S3b). These findings indicate a difference prognosis of cancer patients according to LCN2 expression. In the viewpoint of SAA1 expression, Klüber et al. reported that LCN2 knock out mice showed an increasing mRNA level of SAA1 in the small intestine compared to wild type mice [7]. Yasukawa et al. demonstrated that SAA1 was upregulated in gastric cancer-associated fibroblasts to promote cancer progression [31]. Given the conflicting observations, LCN2 might play a dual role according to cell type, cancer type and organ type. Therefore, further experiments are required to directly address this issue to ensure a better understanding of the interaction of LCN2 with SAA1 in cancer and to figure out the role of SAA1 in GC cells in the future.

In conclusion, LCN2 inhibits EMT signaling through MMP2 downregulation, resulting in the reduction of proliferation, invasion and migration of GC cells. To our knowledge, this study is the first report to clarify the clinical and prognostic significance of LCN2 in GC using public data, as well as our original data. Therefore, LCN2 might be a promising therapeutic target for GC patients with the poor outcome due to the reversal of EMT signaling.

## Supplementary Information

Below is the link to the electronic supplementary material.

Supplementary file1 mRNA levels of LCN2 in gastric cancer cell lines. a LCN2 mRNA level for each GC cell line by RT-PCR. b LCN2 mRNA level in OCUM-12 and NUGC-3 cells was affected by siLCN2. c qPCR analysis of mRNA of LCN2 in OCUM-12 and NUGC-3 cells treated with siLCN2 (PDF 496 KB)

Supplementary file2 RT-PCR for SAA1 expression. a RT-PCR showed degradation of SAA1 mRNA level in OCUM-12 and NUGC-3 cells accompanied by downregulation of LCN2 mRNA levels (PDF 127 KB)

Supplementary file3 The ratio of SAA1 phenotype (a) and genotype (b) in GC patients from GSE113255. These sequence data are visualized with IGV software after genome mapping (PDF 128 KB)

Supplementary file4 a Kaplan–Meier curve for 7-year overall of GC patients from TCGA cohort study according to LCN2 expression (n=415).HR, hazard ratio (PDF 118 KB)

Supplementary file5 (PDF 20 KB)

Supplementary file6 (PDF 79 KB)

Supplementary file7 (PDF 28 KB)

Supplementary file8 (PDF 26 KB)
