# Supplementary figures and images for: Lipocalin-2 negatively regulates epithelial–mesenchymal transition through matrix metalloprotease-2 downregulation in gastric cancer
Source: Gastric Cancer. 2022 Jun 15;25(5):850–61. doi: 10.1007/s10120-022-01305-w (PMC9365736; doi:10.1007/s10120-022-01305-w)

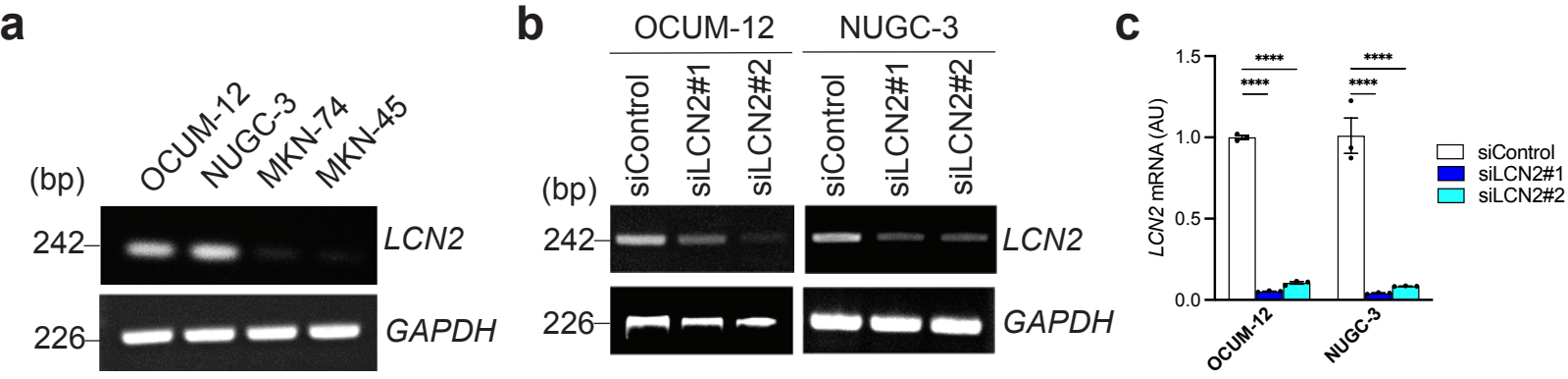

Figure S1

Supplement: Supplementary file 1 — Supplementary file1 mRNA levels of LCN2 in gastric cancer cell lines. a LCN2 mRNA level for each GC cell line by RT-PCR. b LCN2 mRNA level in OCUM-12 and NUGC-3 cells was affected by siLCN2. c qPCR analysis of mRNA of LCN2 in OCUM-12 and NUGC-3 cells treated with siLCN2 (PDF 496 KB) [file 10120_2022_1305_MOESM1_ESM.pdf]

**a**

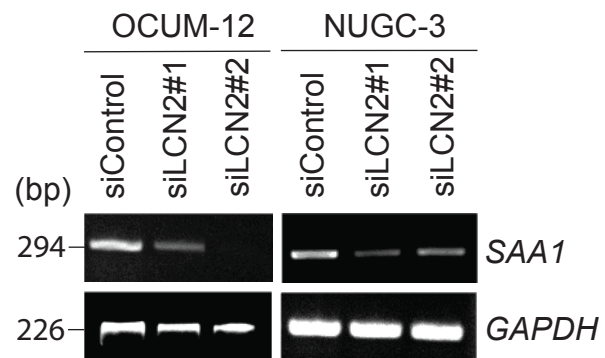

Figure S2

Supplement: Supplementary file 2 — Supplementary file2 RT-PCR for SAA1 expression. a RT-PCR showed degradation of SAA1 mRNA level in OCUM-12 and NUGC-3 cells accompanied by downregulation of LCN2 mRNA levels (PDF 127 KB) [file 10120_2022_1305_MOESM2_ESM.pdf]

**a**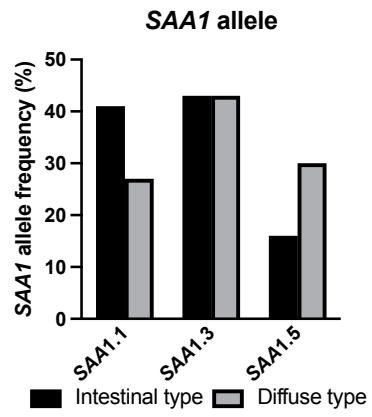**b**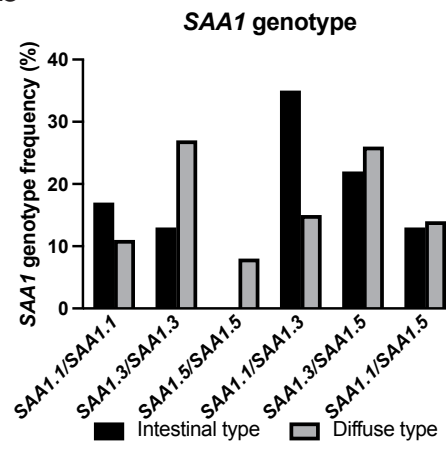

Figure S3

Supplement: Supplementary file 3 — Supplementary file3 The ratio of SAA1 phenotype (a) and genotype (b) in GC patients from GSE113255. These sequence data are visualized with IGV software after genome mapping (PDF 128 KB) [file 10120_2022_1305_MOESM3_ESM.pdf]

**a**

**TCGA cohort study**

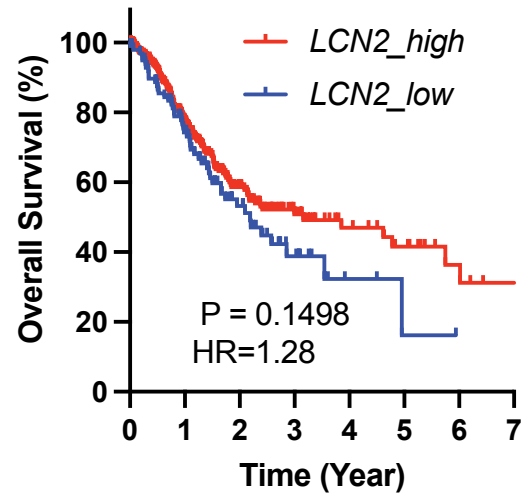

Figure S4

Supplement: Supplementary file 4 — Supplementary file4 a Kaplan–Meier curve for 7-year overall of GC patients from TCGA cohort study according to LCN2 expression (n=415).HR, hazard ratio (PDF 118 KB) [file 10120_2022_1305_MOESM4_ESM.pdf]
