# Supplementary material for: Lipocalin-2 negatively regulates epithelial–mesenchymal transition through matrix metalloprotease-2 downregulation in gastric cancer
Source: Gastric Cancer. 2022 Jun 15;25(5):850–61. doi: 10.1007/s10120-022-01305-w (PMC9365736; doi:10.1007/s10120-022-01305-w)
Supplement: Supplementary file 5 — Supplementary file5 (PDF 20 KB) [file 10120_2022_1305_MOESM5_ESM.pdf]

Table S1 Primers sequences

Gene symbol	Forward	Reverse
LCN2	5'-TCACCTCCGTCCTGTTTAGG-3'	5'-CGAAGTCAGCTCCTTGGTTC-3'
SAA1	5'-CATGCTCGGGGGAACTAT-3'	5'-TACCCATTGTGTACCCTCTC-3'
GAPDH	5'-GAAGGTGAAGGTCGGAGTC-3'	5'-GAAGATGGTGATGGGATTTC-3'
