# Supplementary material for: Lipocalin-2 negatively regulates epithelial–mesenchymal transition through matrix metalloprotease-2 downregulation in gastric cancer
Source: Gastric Cancer. 2022 Jun 15;25(5):850–61. doi: 10.1007/s10120-022-01305-w (PMC9365736; doi:10.1007/s10120-022-01305-w)
Supplement: Supplementary file 6 — Supplementary file6 (PDF 79 KB) [file 10120_2022_1305_MOESM6_ESM.pdf]

Table S2. Complete list of all significantly differential expressed genes between DGC and IGC

Regulation	Ensembl ID	log2 Fold Ch	Adj.Pval	Symbol	Chr	Type
Down	ENSG000001	-2.1047257	3.84E-03	LCN2	9q34.11	protein_coding
Down	ENSG000001	-1.9502125	3.53E-02	DMBT1	10q26.13	protein_coding
Down	ENSG000001	-1.8041682	6.88E-04	SAA1	11p15.1	protein_coding
Down	ENSG000002	-1.7465689	8.10E-03	SLC6A14	Xq23	protein_coding
Down	ENSG000001	-1.6931772	9.73E-03	PI3	20q13.12	protein_coding
Down	ENSG000002	-1.6291219	7.50E-03	MMP12	11q22.2	protein_coding
Down	ENSG000001	-1.5946782	4.45E-04	CXCL3	4q13.3	protein_coding
Down	ENSG000001	-1.5611723	5.60E-03	SLPI	20q13.12	protein_coding
Down	ENSG000001	-1.5202694	6.27E-03	CCL20	2q36.3	protein_coding
Down	ENSG000001	-1.3522304	1.61E-02	S100P	4p16.1	protein_coding
Down	ENSG000002	-1.3297582	9.51E-05	SNORA27	13q12.2	snoRNA
Down	ENSG000001	-1.3238192	6.73E-04	SNORA52	11p15.5	snoRNA
Down	ENSG000002	-1.2969325	1.33E-04	SNORA74A	5q31.2	snoRNA
Down	ENSG000002	-1.2774758	2.38E-03	SNORA68	19p13.11	snoRNA
Down	ENSG000001	-1.2755112	9.36E-03	PCSK9	1p32.3	protein_coding
Down	ENSG000001	-1.2601142	8.63E-04	SAA2	11p15.1	protein_coding
Down	ENSG000001	-1.2433202	1.06E-02	CXCL1	4q13.3	protein_coding
Down	ENSG000001	-1.217113	1.26E-02	VSNL1	2p24.2	protein_coding
Down	ENSG000002	-1.2145055	2.51E-03	SCARNA22	4p16.3	scaRNA
Down	ENSG000002	-1.193887	3.55E-03	SNORA70	Xq28	snoRNA
Down	ENSG000001	-1.1876217	2.06E-02	CXCL11	4q21.1	protein_coding
Down	ENSG000001	-1.1858346	1.29E-03	PSAT1	9q21.2	protein_coding
Down	ENSG000001	-1.1765567	1.19E-02	PDZK1IP1	1p33	protein_coding
Down	ENSG000002	-1.1756717	1.73E-04	SCARNA1	1p35.3	scaRNA
Down	ENSG000001	-1.1627017	6.99E-03	TMPRSS3	21q22.3	protein_coding
Down	ENSG000002	-1.1431703	4.41E-03	SNORD97	11p15.4	snoRNA
Down	ENSG000002	-1.1338525	4.68E-03	SNORA71A	20q11.23	snoRNA
Down	ENSG000002	-1.1287481	1.06E-05	SNORA40	11q21	snoRNA
Down	CXORF61	-1.1042674	1.13E-02	NA	NANA	NA

Down	ENSG000002	-1.084054	2.85E-02	CCL18	17q12	protein_coding
Down	ENSG000002	-1.0812007	2.28E-03	SNORA37	18q21.2	snoRNA
Down	ENSG000002	-1.0806071	8.21E-04	RNU6ATAC	9q34.2	snRNA
Down	SNORA76	-1.0798257	8.44E-04	NA	NANA	NA
Down	ENSG000002	-1.0727733	1.88E-06	SNORA18	11q21	snoRNA
Down	GPR110	-1.0708147	3.28E-02	NA	NANA	NA
Down	SNORA3	-1.0639289	4.01E-04	NA	NANA	NA
Down	ENSG000002	-1.0547785	5.66E-03	SNORA64	16p13.3	snoRNA
Down	ENSG000001	-1.0478919	2.42E-02	HRASLS2	11q12.3	protein_coding
Down	ENSG000001	-1.0400053	1.05E-03	CCNE1	19q12	protein_coding
Down	ENSG000002	-1.0380829	3.01E-04	SNORD22	11q12.3	snoRNA
Down	ENSG000001	-1.0292406	1.16E-02	C6orf223	6p21.1	protein_coding
Down	ENSG000001	-1.0151333	4.08E-02	MUC1	1q22	protein_coding
Up	ENSG000002	1.00057172	3.67E-04	ERVK13-1	16p13.3	processed_transcript
Up	ENSG000001	1.00067818	4.45E-03	PLCXD3	5p13.1	protein_coding
Up	ENSG000001	1.00102357	1.54E-03	ZSCAN18	19q13.43	protein_coding
Up	LOC1005056	1.0016989	9.11E-04	NA	NANA	NA
Up	ENSG000001	1.00199878	7.55E-02	A1CF	10q11.23	protein_coding
Up	ENSG000001	1.00288541	4.95E-04	SV2B	15q26.1	protein_coding
Up	ENSG000002	1.00387566	1.08E-04	GSTM2	1p13.3	protein_coding
Up	ENSG000002	1.00428525	2.24E-03	MIR612	11q13.1	miRNA
Up	GPR64	1.00504673	1.54E-03	NA	NANA	NA
Up	ENSG000002	1.00577976	7.34E-04	FXYD1	19q13.12	protein_coding
Up	MATL2963	1.00608127	6.24E-04	NA	NANA	NA
Up	ENSG000001	1.00857822	4.51E-03	TCF21	6q23.2	protein_coding
Up	ENSG000001	1.0090768	3.15E-03	MAP1B	5q13.2	protein_coding
Up	ENSG000001	1.01100975	4.19E-03	PDZRN3	3p13	protein_coding
Up	ENSG000001	1.01245469	5.75E-03	TOX	8q12.1	protein_coding
Up	ENSG000001	1.01285697	1.13E-03	CNTNAP1	17q21.2	protein_coding
Up	ENSG000001	1.01397237	1.14E-03	LTBP3	11q13.1	protein_coding
Up	ENSG000001	1.0140061	8.73E-05	IL11RA	9p13.3	protein_coding

Up	ENSG000001	1.01540228	2.16E-02	FSIP2	2q32.1	protein_coding
Up	ENSG000002	1.01693824	1.12E-03	RNF138P1	5q11.2	processed_pseudogene
Up	ENSG000001	1.01850183	1.38E-04	CACNB4	2q23.3	protein_coding
Up	ENSG00000C	1.01892076	7.01E-03	CRLF1	19p13.11	protein_coding
Up	ENSG000001	1.02002235	6.90E-03	SPARCL1	4q22.1	protein_coding
Up	LOC388630	1.02102641	2.51E-03	NA	NANA	NA
Up	ENSG000001	1.02260585	1.04E-02	SRPX	Xp11.4	protein_coding
Up	ENSG000001	1.0226664	7.33E-03	CACNA1E	1q25.3	protein_coding
Up	ENSG000001	1.02302357	5.11E-03	SMAD9	13q13.3	protein_coding
Up	ODZ4	1.02332995	6.28E-03	NA	NANA	NA
Up	ENSG000001	1.02352824	8.42E-03	NBEA	13q13.3	protein_coding
Up	ENSG000001	1.02444535	5.31E-03	AEBP1	7p13	protein_coding
Up	ENSG000001	1.02479724	3.04E-02	PCSK1	5q15	protein_coding
Up	ENSG00000C	1.02508614	3.52E-04	PDZD4	Xq28	protein_coding
Up	ENSG000001	1.02550711	8.21E-04	SHC2	19p13.3	protein_coding
Up	ENSG000001	1.02744819	3.90E-04	MAP3K12	12q13.13	protein_coding
Up	ENSG000001	1.02750752	2.68E-05	C9orf131	9p13.3	protein_coding
Up	ENSG000001	1.02771556	3.16E-03	TIMP3	22q12.3	protein_coding
Up	ENSG000001	1.02785291	1.48E-03	EGR1	5q31.2	protein_coding
Up	ENSG000001	1.02985616	1.32E-02	HAND2	4q34.1	protein_coding
Up	ENSG000001	1.0309874	4.45E-04	SALL2	14q11.2	protein_coding
Up	ENSG000001	1.03109671	6.43E-02	FNDC1	6q25.3	protein_coding
Up	ENSG000002	1.03286875	8.60E-03	KCNQ1OT1	11p15.5	antisense
Up	ENSG000001	1.03482324	2.37E-02	IGFN1	1q32.1	protein_coding
Up	ENSG000001	1.03507436	5.97E-04	EVC2	4p16.2	protein_coding
Up	ENSG000001	1.03635392	5.08E-04	LENG8	19q13.42	protein_coding
Up	ENSG000001	1.03651483	3.53E-03	PLEKHH2	2p21	protein_coding
Up	LOC1001315	1.03752174	3.02E-04	NA	NANA	NA
Up	ENSG000001	1.03962779	1.27E-02	SLIT3	5q35.1	protein_coding
Up	PEG3	1.03967249	1.51E-03	NA	NANA	NA
Up	ENSG000001	1.04109061	3.44E-04	CDH23	10q22.1	protein_coding

Up	ENSG000001	1.04170419	6.36E-03	SCG2	2q36.1	protein_coding
Up	ENSG000001	1.04227672	1.46E-03	DSEL	18q22.1	protein_coding
Up	ENSG000001	1.04286306	1.26E-03	ZFPM2	8q22.3	protein_coding
Up	ENSG000001	1.04587972	1.99E-03	THSD7B	2q22.1	protein_coding
Up	ENSG000001	1.04622796	2.22E-03	NALCN	13q33.1	protein_coding
Up	ENSG00000C	1.04680699	3.95E-03	MYLK	3q21.1	protein_coding
Up	ENSG000001	1.0469167	1.90E-03	AUTS2	7q11.22	protein_coding
Up	ENSG000001	1.04726006	3.57E-02	AGT	1q42.2	protein_coding
Up	ENSG000001	1.04945429	1.09E-02	ANO7	2q37.3	protein_coding
Up	ENSG00000C	1.05026168	6.93E-03	RIMS1	6q13	protein_coding
Up	ENSG000002	1.05035799	6.26E-03	INMT	7p14.3	protein_coding
Up	ENSG000001	1.05096953	3.37E-03	PTCH1	9q22.32	protein_coding
Up	ENSG000001	1.05349655	1.52E-03	OBSL1	2q35	protein_coding
Up	ENSG000001	1.05616782	3.13E-03	TSHZ2	20q13.2	protein_coding
Up	ENSG000001	1.05632954	4.93E-03	C2orf40	2q12.2	protein_coding
Up	ENSG000001	1.05680171	9.89E-05	CAND2	3p25.2	protein_coding
Up	ENSG000001	1.057141	4.58E-04	ZNF512B	20q13.33	protein_coding
Up	ENSG000001	1.05840959	1.28E-03	RIMKLB	12p13.31	protein_coding
Up	ENSG000001	1.05856522	1.91E-03	MSRB3	12q14.3	protein_coding
Up	ENSG000001	1.05923161	3.62E-03	RERG	12p12.3	protein_coding
Up	ENSG000001	1.06021577	1.07E-03	MPDZ	9p23	protein_coding
Up	C1ORF95	1.06042097	1.07E-03	NA	NANA	NA
Up	ENSG000001	1.06057416	1.21E-04	NLGN3	Xq13.1	protein_coding
Up	ENSG00000C	1.06076676	1.30E-02	TRHDE	12q21.1	protein_coding
Up	ENSG000001	1.06198944	7.35E-04	GEM	8q22.1	protein_coding
Up	ENSG000001	1.06206501	1.11E-02	FBLN2	3p25.1	protein_coding
Up	ENSG00000C	1.06259813	3.23E-04	FAT2	5q33.1	protein_coding
Up	ENSG000001	1.06785006	4.77E-02	FREM2	13q13.3	protein_coding
Up	ENSG00000C	1.06841528	8.21E-04	NLRP1	17p13.2	protein_coding
Up	ENSG000001	1.06902438	1.01E-03	PRIMA1	14q32.12	protein_coding
Up	ENSG000001	1.06937302	6.07E-04	SAMD11	1p36.33	protein_coding

Up	ENSG00000C	1.06956603	8.95E-04	LIMS2	2q14.3	protein_coding
Up	ENSG000001	1.06984681	2.03E-02	THBS2	6q27	protein_coding
Up	ENSG00000C	1.07162983	2.94E-03	SORBS1	10q24.1	protein_coding
Up	ENSG00000C	1.07175985	5.77E-04	CHRD	3q27.1	protein_coding
Up	ENSG000001	1.07320033	2.99E-03	AKAP6	14q12	protein_coding
Up	ENSG000001	1.07583462	1.75E-03	HSPB8	12q24.23	protein_coding
Up	ENSG00000C	1.07722349	1.55E-04	CAPN3	15q15.1	protein_coding
Up	ENSG000001	1.07789232	1.82E-02	TPH1	11p15.1	protein_coding
Up	ENSG00000C	1.0784104	4.82E-03	KCNH2	7q36.1	protein_coding
Up	ENSG000001	1.07909427	1.13E-03	RNF150	4q31.21	protein_coding
Up	ENSG000002	1.08039903	1.49E-03	FCGR2C	1q23.3	polymorphic_pseudogene
Up	ENSG000001	1.08148395	9.89E-05	ZBTB20	3q13.31	protein_coding
Up	ENSG000001	1.08248111	2.23E-03	TPM2	9p13.3	protein_coding
Up	ENSG000001	1.08268468	7.22E-02	CA9	9p13.3	protein_coding
Up	ENSG000001	1.0831731	7.31E-04	SYNE1	6q25.2	protein_coding
Up	ENSG000001	1.08762414	1.44E-03	PDE1A	2q32.1	protein_coding
Up	ENSG00000C	1.08838968	1.05E-02	FER1L4	20q11.22	transcribed_unitary_pseudogene
Up	ENSG00000C	1.08982324	1.18E-02	SEMA6A	5q23.1	protein_coding
Up	ENSG000001	1.08986835	3.84E-04	ARMCX4	Xq22.1	protein_coding
Up	ENSG000001	1.0906156	2.37E-03	MYL9	20q11.23	protein_coding
Up	ODZ2	1.09116172	5.68E-03	NA	NANA	NA
Up	ENSG000001	1.09197684	9.99E-03	FGF7	15q21.2	protein_coding
Up	ENSG000001	1.09308533	3.20E-03	GHRL	3p25.3	protein_coding
Up	ENSG000001	1.10048232	4.75E-04	BHMT2	5q14.1	protein_coding
Up	ENSG000001	1.10112515	1.47E-02	CAPN13	2p23.1	protein_coding
Up	ENSG000001	1.10136083	3.18E-03	MPV17L	16p13.11	protein_coding
Up	ENSG000001	1.10337586	3.60E-03	LGI2	4p15.2	protein_coding
Up	ENSG000002	1.10470419	4.51E-04	FBXO17	19q13.2	protein_coding
Up	ENSG000001	1.10585291	1.72E-02	IL33	9p24.1	protein_coding
Up	SDPR	1.10657822	3.21E-03	NA	NANA	NA
Up	ENSG000001	1.11217188	2.21E-03	ZNF423	16q12.1	protein_coding

Up	ENSG000001	1.11364242	2.71E-04	DLG2	11q14.1	protein_coding
Up	ENSG000001	1.11388094	5.73E-03	RSPO3	6q22.33	protein_coding
Up	ENSG000000	1.11428891	7.03E-04	CLDN11	3q26.2	protein_coding
Up	ENSG000001	1.11453677	2.64E-03	MRGPRF	11q13.3	protein_coding
Up	PER1	1.11582202	4.51E-04	NA	NANA	NA
Up	ENSG000001	1.11761113	4.42E-03	LONRF2	2q11.2	protein_coding
Up	ENSG000001	1.1187631	1.05E-03	AFF3	2q11.2	protein_coding
Up	ENSG000001	1.11998172	7.23E-04	PRTG	15q21.3	protein_coding
Up	ENSG000000	1.12018895	1.81E-03	GLI2	2q14.2	protein_coding
Up	ENSG000001	1.12063795	2.77E-02	SYNM	15q26.3	protein_coding
Up	ENSG000001	1.12095287	3.23E-04	DTX1	12q24.13	protein_coding
Up	ENSG000001	1.12200935	4.16E-04	DMPK	19q13.32	protein_coding
Up	ENSG000001	1.12273913	7.03E-04	ITGA7	12q13.2	protein_coding
Up	ENSG000001	1.12667615	1.87E-03	CMYA5	5q14.1	protein_coding
Up	ENSG000001	1.12718814	9.52E-04	PDE7B	6q23.3	protein_coding
Up	ENSG000001	1.12854124	5.02E-03	CGNL1	15q21.3	protein_coding
Up	ENSG000001	1.13117473	6.76E-02	SFRP4	7p14.1	protein_coding
Up	ENSG000001	1.1321028	1.29E-03	NR2F1	5q15	protein_coding
Up	ENSG000001	1.13243681	2.24E-03	ALDH1A3	15q26.3	protein_coding
Up	PAR5	1.13316132	4.22E-04	NA	NANA	NA
Up	ENSG000001	1.13443681	4.26E-03	JPH2	20q13.12	protein_coding
Up	ENSG000000	1.13448111	7.11E-03	ZFHX4	8q21.13	protein_coding
Up	ENSG000001	1.13454124	2.68E-04	ID4	6p22.3	protein_coding
Up	ENSG000001	1.13635189	3.84E-04	KCNT2	1q31.3	protein_coding
Up	ENSG000000	1.13682487	9.11E-04	PTGER3	1p31.1	protein_coding
Up	ENSG000002	1.1375831	1.95E-03	C14orf132	14q32.2	protein_coding
Up	ENSG000001	1.13761723	1.25E-03	ACTA2	10q23.31	protein_coding
Up	ENSG000001	1.13778586	3.94E-03	CPXM2	10q26.13	protein_coding
Up	ENSG000001	1.13860463	1.55E-03	MAP1A	15q15.3	protein_coding
Up	ENSG000001	1.13964039	4.58E-03	EFEMP1	2p16.1	protein_coding
Up	ENSG000001	1.14005811	6.36E-03	RELN	7q22.1	protein_coding

Up	ENSG000001	1.14163592	1.03E-02	AOX1	2q33.1	protein_coding
Up	ENSG00000C	1.14249045	6.71E-03	SPEG	2q35	protein_coding
Up	ENSG000001	1.14289435	9.64E-04	PTPRS	19p13.3	protein_coding
Up	ENSG000001	1.14367493	3.44E-02	PCLO	7q21.11	protein_coding
Up	ENSG000001	1.14425884	1.21E-04	TTC28	22q12.1	protein_coding
Up	TENC1	1.14506014	3.23E-04	NA	NANA	NA
Up	ENSG000001	1.14598131	1.55E-03	RBMS3	3p24.1	protein_coding
Up	ENSG000001	1.14632304	8.52E-03	OMD	9q22.31	protein_coding
Up	ENSG000001	1.14988623	2.80E-03	ITIH5	10p14	protein_coding
Up	ENSG000001	1.15064974	1.65E-03	HEYL	1p34.2	protein_coding
Up	ENSG000001	1.15254531	3.18E-04	CACNB2	10p12.33	protein_coding
Up	ENSG000001	1.15294311	1.12E-03	DNM1	9q34.11	protein_coding
Up	ENSG000001	1.15471556	6.07E-04	ARHGEF26	3q25.2	protein_coding
Up	ENSG00000C	1.15480699	8.08E-03	SLC7A2	8p22	protein_coding
Up	ENSG000001	1.15531694	2.94E-03	SCARA3	8p21.1	protein_coding
Up	ENSG000001	1.15651158	1.69E-03	PDE3A	12p12.2	protein_coding
Up	ENSG000001	1.16079521	1.11E-03	FAM107A	3p14.2	protein_coding
Up	ENSG000001	1.16116416	1.02E-02	SHISA3	4p13	protein_coding
Up	ENSG000001	1.16127387	3.13E-03	WNT2B	1p13.2	protein_coding
Up	ENSG000001	1.16342747	4.76E-04	ADAMTS10	19p13.2	protein_coding
Up	ENSG000001	1.16683665	2.28E-03	DPYSL3	5q32	protein_coding
Up	ENSG000001	1.16811743	2.96E-02	GALNT8	12p13.32	protein_coding
Up	ENSG00000C	1.17244738	6.73E-04	EVC	4p16.2	protein_coding
Up	ENSG000002	1.1726924	5.55E-05	TP73-AS1	1p36.32	transcribed_unitary_pseudogene
Up	ENSG000001	1.17608655	2.03E-04	ZNF483	9q31.3	protein_coding
Up	ENSG00000C	1.17693539	5.99E-04	GGT5	22q11.23	protein_coding
Up	DARC	1.17834539	9.62E-03	NA	NANA	NA
Up	ENSG000001	1.17957334	3.20E-03	CDR1	Xq27.1	protein_coding
Up	ENSG000001	1.18007761	4.85E-03	FOXP2	7q31.1	protein_coding
Up	ENSG000001	1.18098618	6.46E-04	NFATC4	14q12	protein_coding
Up	ENSG000001	1.18131938	7.03E-04	SCN4B	11q23.3	protein_coding

Up	ENSG000001	1.18256278	4.03E-03	PCDH9	13q21.32	protein_coding
Up	ENSG000001	1.18280333	8.26E-04	DYNC2H1	11q22.3	protein_coding
Up	ENSG000001	1.18518529	3.84E-04	CRYAB	11q23.1	protein_coding
Up	ENSG000002	1.18795774	6.73E-04	HSPA7	1q23.3	unprocessed_pseudogene
Up	ENSG00000C	1.18824868	2.11E-03	RUNX1T1	8q21.3	protein_coding
Up	ENSG000001	1.19115969	4.91E-03	RYR2	1q43	protein_coding
Up	ENSG000001	1.19149858	3.18E-04	TUB	11p15.4	protein_coding
Up	GALNTL1	1.19453556	1.48E-03	NA	NANA	NA
Up	ENSG000001	1.19470947	8.47E-05	KCND3	1p13.2	protein_coding
Up	ENSG000001	1.19998293	8.18E-04	MAPK10	4q21.3	protein_coding
Up	ENSG000001	1.20117188	3.84E-04	ADAMTSL4	1q21.2	protein_coding
Up	ENSG000001	1.20166396	2.22E-03	IGSF10	3q25.1	protein_coding
Up	ENSG00000C	1.21274198	6.26E-03	CNTN1	12q12	protein_coding
Up	ENSG000001	1.21462211	1.99E-03	C1QTNF7	4p15.32	protein_coding
Up	ENSG00000C	1.21466396	1.81E-04	ACACB	12q24.11	protein_coding
Up	ENSG000001	1.21680415	8.22E-02	HEPACAM2	7q21.2	protein_coding
Up	ENSG000001	1.21968184	8.47E-03	SVEP1	9q31.3	protein_coding
Up	ENSG000001	1.22311784	2.21E-02	GPC3	Xq26.2	protein_coding
Up	ENSG000001	1.22326209	2.25E-04	DNAH1	3p21.1	protein_coding
Up	ENSG000001	1.22793092	7.03E-04	SLC24A3	20p11.23	protein_coding
Up	ENSG000001	1.22904957	1.05E-02	TYRP1	9p23	protein_coding
Up	ENSG000001	1.23183421	6.59E-03	MFAP5	12p13.31	protein_coding
Up	ENSG000001	1.23248111	2.16E-02	ITGBL1	13q33.1	protein_coding
Up	ENSG000001	1.2334677	3.52E-04	DDR2	1q23.3	protein_coding
Up	ENSG00000C	1.23522024	1.93E-03	LMO3	12p12.3	protein_coding
Up	ENSG000001	1.23541528	1.30E-03	SCRG1	4q34.1	protein_coding
Up	ENSG000001	1.23732263	1.08E-02	XPNPEP2	Xq26.1	protein_coding
Up	ENSG000001	1.23945022	3.71E-02	ADH4	4q23	protein_coding
Up	ENSG00000C	1.24208371	1.65E-03	ABCC9	12p12.1	protein_coding
Up	C11ORF92	1.24435799	8.10E-03	NA	NANA	NA
Up	ENSG000001	1.25331126	7.11E-03	NRK	Xq22.3	protein_coding

Up	LOC148696	1.25419829	6.79E-05	NA	NANA	NA
Up	ENSG00000C	1.25714588	7.34E-04	MUSK	9q31.3	protein_coding
Up	ENSG00000C	1.25715563	3.55E-04	PALM	19p13.3	protein_coding
Up	ENSG000001	1.26335677	1.72E-04	PHLDB2	3q13.2	protein_coding
Up	ENSG000001	1.26391223	2.76E-02	SFRP1	8p11.21	protein_coding
Up	ENSG000001	1.26410118	1.99E-03	BGN	Xq28	protein_coding
Up	ENSG000001	1.26423852	1.00E-03	VIPR2	7q36.3	protein_coding
Up	ENSG000001	1.26721983	1.78E-03	PLN	6q22.31	protein_coding
Up	ENSG000001	1.26935189	3.84E-04	GLI1	12q13.3	protein_coding
Up	ENSG000002	1.27286144	1.21E-04	ARHGEF25	12q13.3	protein_coding
Up	ENSG00000C	1.27645998	4.38E-02	DPEP1	16q24.3	protein_coding
Up	ENSG000001	1.27702763	4.95E-04	FMOD	1q32.1	protein_coding
Up	ENSG000001	1.28033686	6.24E-04	STARD9	15q15.2	protein_coding
Up	ENSG000001	1.28480415	4.45E-04	NRXN2	11q13.1	protein_coding
Up	C5ORF4	1.28785047	1.21E-04	NA	NANA	NA
Up	ENSG000001	1.29178627	3.03E-03	ABI3BP	3q12.2	protein_coding
Up	ENSG000001	1.29316254	3.31E-03	C16orf89	16p13.3	protein_coding
Up	ENSG00000C	1.30097237	2.65E-03	FMO2	1q24.3	protein_coding
Up	ENSG000002	1.30327062	5.51E-03	SPON1	11p15.2	protein_coding
Up	ENSG000002	1.30650508	1.59E-03	PLXNA4	7q32.3	protein_coding
Up	ENSG000001	1.31485453	3.30E-03	COL8A1	3q12.1	protein_coding
Up	ENSG000001	1.31786428	2.24E-03	DCLK1	13q13.3	protein_coding
Up	ENSG000001	1.32267005	2.42E-03	PODN	1p32.3	protein_coding
Up	ENSG000001	1.32296668	8.44E-04	MAMDC2	9q21.12	protein_coding
Up	ENSG000001	1.32369728	7.30E-04	THSD4	15q23	protein_coding
Up	ENSG000001	1.32490451	5.65E-03	MFAP4	17p11.2	protein_coding
Up	ENSG000001	1.32958513	5.12E-04	PDLIM3	4q35.1	protein_coding
Up	ENSG000001	1.33040796	8.92E-04	DMD	Xp21.1	protein_coding
Up	ENSG00000C	1.33416497	1.96E-03	FHL1	Xq26.3	protein_coding
Up	ENSG000001	1.3348269	1.38E-03	SSC5D	19q13.42	protein_coding
Up	ENSG00000C	1.34276798	2.83E-03	EPHA3	3p11.1	protein_coding

Up	ENSG000001	1.34298456	5.50E-03	CCL19	9p13.3	protein_coding
Up	ENSG00000C	1.34407355	5.56E-04	TNS1	2q35	protein_coding
Up	ENSG000001	1.34936286	1.89E-04	GPRASP1	Xq22.1	protein_coding
Up	ENSG000001	1.3511536	8.18E-04	MN1	22q12.1	protein_coding
Up	ENSG000001	1.36647095	4.45E-04	SETBP1	18q12.3	protein_coding
Up	ENSG000001	1.36653515	1.87E-03	ABCA10	17q24.3	protein_coding
Up	ENSG000001	1.36865177	2.98E-02	APOD	3q29	protein_coding
Up	ENSG00000C	1.37318488	4.81E-04	GNAO1	16q13	protein_coding
Up	ENSG000001	1.37475173	9.76E-04	LAMA2	6q22.33	protein_coding
Up	ENSG000001	1.37730191	1.46E-02	PTGDS	9q34.3	protein_coding
Up	ENSG000001	1.3792113	4.41E-03	GREM1	15q13.3	protein_coding
Up	ENSG00000C	1.38174523	5.52E-04	NGFR	17q21.33	protein_coding
Up	ENSG000001	1.38647582	3.84E-03	AHNAK2	14q32.33	protein_coding
Up	ENSG000002	1.39030394	5.68E-04	MIR100HG	11q24.1	processed_transcript
Up	ENSG000001	1.39582893	5.10E-04	COL4A3	2q36.3	protein_coding
Up	ENSG000001	1.39691182	9.14E-04	ADAMTSL3	15q25.2	protein_coding
Up	LINC00478	1.39735067	6.46E-04	NA	NANA	NA
Up	ENSG000001	1.39761357	2.27E-03	CLU	8p21.1	protein_coding
Up	ENSG000001	1.39816619	1.17E-03	HSPB7	1p36.13	protein_coding
Up	ENSG000001	1.39843478	7.45E-04	BNC2	9p22.2	protein_coding
Up	ENSG000001	1.39901788	8.92E-04	ABCA6	17q24.3	protein_coding
Up	ENSG000001	1.40273425	1.55E-04	ANGPTL1	1q25.2	protein_coding
Up	ENSG000001	1.40896221	9.55E-04	PLIN4	19p13.3	protein_coding
Up	ENSG00000C	1.41387525	1.66E-03	HSPB6	19q13.12	protein_coding
Up	ENSG000001	1.4159297	5.26E-02	ANPEP	15q26.1	protein_coding
Up	ENSG000001	1.42182527	5.69E-03	MGP	12p12.3	protein_coding
Up	ENSG00000C	1.42297684	3.94E-03	ELN	7q11.23	protein_coding
Up	ENSG000001	1.42507355	8.68E-04	MYOCD	17p12	protein_coding
Up	ENSG000001	1.44080699	6.40E-04	TAGLN	11q23.3	protein_coding
Up	ENSG00000C	1.44528484	6.03E-03	IGF1	12q23.2	protein_coding
Up	ENSG000001	1.4492369	8.62E-04	ANK2	4q25	protein_coding

Up	ENSG000001	1.44926371	4.34E-03	PTGIS	20q13.13	protein_coding
Up	ENSG000001	1.461551	8.85E-04	HHIP	4q31.21	protein_coding
Up	ENSG000002	1.47200488	8.85E-04	MEG3	14q32.2	lincRNA
Up	ENSG000001	1.47593011	7.51E-04	TNN	1q25.1	protein_coding
Up	ENSG000001	1.4801922	1.02E-03	ZBTB16	11q23.2	protein_coding
Up	ENSG000000	1.48161113	7.64E-04	NAV3	12q21.2	protein_coding
Up	ENSG000001	1.48918935	9.21E-04	FLRT2	14q31.3	protein_coding
Up	ENSG000001	1.49152702	3.48E-04	ADCY5	3q21.1	protein_coding
Up	ENSG000000	1.50913653	3.45E-03	CCDC80	3q13.2	protein_coding
Up	ENSG000001	1.50980415	1.11E-04	AOC3	17q21.31	protein_coding
Up	ENSG000001	1.51003048	7.03E-04	PGM5	9q21.11	protein_coding
Up	ENSG000001	1.53476717	5.12E-04	MATN2	8q22.1	protein_coding
Up	FAM198A	1.53647501	6.68E-04	NA	NANA	NA
Up	ENSG000001	1.54134905	4.27E-04	NFASC	1q32.1	protein_coding
Up	ENSG000001	1.54139781	6.79E-05	AKAP12	6q25.1	protein_coding
Up	ENSG000001	1.54368996	5.52E-04	ABCA9	17q24.2	protein_coding
Up	ENSG000001	1.54525762	1.45E-03	ADAM33	20p13	protein_coding
Up	ENSG000001	1.54761438	1.05E-03	SLIT2	4p15.31	protein_coding
Up	ENSG000001	1.54957375	8.08E-03	PRUNE2	9q21.2	protein_coding
Up	ENSG000001	1.55337586	2.69E-03	GFRA1	10q25.3	protein_coding
Up	ENSG000001	1.55523121	1.65E-03	FOSB	19q13.32	protein_coding
Up	ENSG000000	1.55757456	4.37E-03	FBLN1	22q13.31	protein_coding
Up	ENSG000001	1.57080577	8.21E-04	CYP1B1	2p22.2	protein_coding
Up	GPR133	1.5810772	5.38E-04	NA	NANA	NA
Up	ENSG000000	1.58539781	1.48E-03	COL4A4	2q36.3	protein_coding
Up	ENSG000001	1.59403942	6.40E-04	BOC	3q13.2	protein_coding
Up	ENSG000001	1.60918529	2.25E-03	HMCN1	1q25.3	protein_coding
Up	ENSG000001	1.60956441	5.30E-03	ACTG2	2p13.1	protein_coding
Up	ENSG000001	1.61517676	3.02E-04	SMOC2	6q27	protein_coding
Up	ENSG000000	1.61900041	7.84E-04	CHRDL2	11q13.4	protein_coding
Up	ENSG000001	1.62692767	5.78E-03	CHRDL1	Xq23	protein_coding

Up	ENSG000001	1.62777895	7.94E-04	LMOD1	1q32.1	protein_coding
Up	ENSG00000C	1.64359651	7.30E-03	CAPN6	Xq23	protein_coding
Up	ENSG000001	1.64583543	1.26E-02	ADH1B	4q23	protein_coding
Up	ENSG000001	1.65053515	7.45E-04	RGMA	15q26.1	protein_coding
Up	ENSG000001	1.70419342	1.66E-03	GREM2	1q43	protein_coding
Up	ENSG000001	1.71726087	3.07E-03	ABCA8	17q24.2	protein_coding
Up	ENSG000001	1.72817798	1.29E-03	SYNPO2	4q26	protein_coding
Up	ENSG000001	1.73073954	1.30E-03	CNN1	19p13.2	protein_coding
Up	ENSG000001	1.78084275	5.72E-04	SCN7A	2q24.3	protein_coding
Up	ENSG000001	1.79729378	7.96E-04	PRELP	1q32.1	protein_coding
Up	ENSG000001	1.79985412	6.19E-03	SFRP2	4q31.3	protein_coding
Up	ENSG000001	1.80625559	8.00E-04	COL14A1	8q24.12	protein_coding
Up	ENSG000001	1.88490492	3.52E-04	FLNC	7q32.1	protein_coding
Up	MIR143HG	1.89851768	2.06E-04	NA	NANA	NA
Up	ENSG000001	1.94123324	1.89E-03	CILP	15q22.31	protein_coding
Up	ENSG000001	2.03431126	1.25E-02	HMGCS2	1p12	protein_coding
Up	ENSG000001	2.04565624	1.65E-03	OGN	9q22.31	protein_coding
Up	ENSG000001	2.10932954	3.52E-04	FREM1	9p22.3	protein_coding
Up	ENSG000001	2.19719057	4.45E-04	MYH11	16p13.11	protein_coding
Up	ENSG000001	2.47022186	7.96E-03	MUC6	11p15.5	protein_coding
Up	ENSG000001	2.51686266	1.59E-03	C7	5p13.1	protein_coding
Up	ENSG000001	2.52113369	2.92E-03	DES	2q35	protein_coding
Up	ENSG000001	2.86430841	6.73E-04	THBS4	5q14.1	protein_coding
