# Supplementary material for: Lipocalin-2 negatively regulates epithelial–mesenchymal transition through matrix metalloprotease-2 downregulation in gastric cancer
Source: Gastric Cancer. 2022 Jun 15;25(5):850–61. doi: 10.1007/s10120-022-01305-w (PMC9365736; doi:10.1007/s10120-022-01305-w)
Supplement: Supplementary file 7 — Supplementary file7 (PDF 28 KB) [file 10120_2022_1305_MOESM7_ESM.pdf]

Table S3. Patient demographics in GC patients from ACRG cohort study according to LCN2 mRNA level

Variables		LCN2 mRNA level High n=229 (%)	LCN2 mRNA level Low n=63 (%)	p.value
Age		64.00 [56.00, 70.00]*	62.00 [53.00, 69.50]	0.274
Sex	Female	80 (34.9)	19 (30.2)	0.549
	Male	149 (65.1)	44 (69.8)	
Lauren Classification	Intestinal	128 (55.9)	22 (34.9)	0.004
	Diffuse	101 (44.1)	41 (65.1)	
T_factor(%)	T1/2	156 (68.1)	27 (42.9)	<0.001
	T3/4	73 (31.9)	36 (57.1)	
N factor (%)	Negative	28 (12.2)	8 (12.7)	1
	Positive	201 (87.8)	55 (87.3)	
M factor(%)	Negative	207 (90.4)	58 (92.1)	0.809
	Positive	22 (9.6)	5 (7.9)	
Lymphatic invasion	Negative	60 (27.9)	12 (21.8)	0.398
	Positive	155 (72.1)	43 (78.2)	
Venous Invasion (%)	Negative	99 (73.3)	24 (75.0)	1
	Positive	36 (26.7)	8 (25.0)	
Perineural.Invasion	Negative	129 (66.8)	26 (54.2)	0.129
	Positive	64 (33.2)	22 (45.8)	
Helicobactor Pylori infection	Negative	56 (58.3)	15 (51.7)	0.531
	Positive	40 (41.7)	14 (48.3)	

*Values are median [interquartile range].
