# Supplementary material for: Lipocalin-2 negatively regulates epithelial–mesenchymal transition through matrix metalloprotease-2 downregulation in gastric cancer
Source: Gastric Cancer. 2022 Jun 15;25(5):850–61. doi: 10.1007/s10120-022-01305-w (PMC9365736; doi:10.1007/s10120-022-01305-w)
Supplement: Supplementary file 8 — Supplementary file8 (PDF 26 KB) [file 10120_2022_1305_MOESM8_ESM.pdf]

Table S4. Correlation between the expression of LCN2 and clinicopathologic features in 590 patients with GC

Vriables		LCN2 expression		
		High n=366 (%)	Low n=224 (%)	p.value
Age	<65	158 (43.2)	103 (46.0)	0.56
	65<	208 (56.8)	121 (54.0)	
Sex	Female	157 (42.9)	103 (46.0)	0.517
	Male	209 (57.1)	121 (54.0)	
Histologic type	Differentiated	205 (56.0)	82 (36.6)	<0.001
	Undifferentiated	161 (44.0)	142 (63.4)	
T status	T1/2	235 (64.2)	104 (46.4)	<0.001
	T3/4	131 (35.8)	120 (53.6)	
Nodal involvement	Negative	224 (61.2)	106 (48.0)	0.002
	Positive	142 (38.8)	115 (52.0)	
M factor	Negative	363 (99.2)	210 (93.8)	<0.001
	Positive	3 (0.8)	14 (6.2)	
Lymphatic invasion	Negative	173 (47.4)	88 (39.3)	0.066
	Positive	192 (52.6)	136 (60.7)	
Venous invasion	Negative	302 (82.5)	184 (82.1)	0.997
	Positive	64 (17.5)	40 (17.9)	
